# Metformin modifies disparity in hepatocellular carcinoma incidence in men with type 2 diabetes but without chronic liver diseases

**DOI:** 10.1002/cam4.2142

**Published:** 2019-04-16

**Authors:** Chen‐Pin Wang, John Kuhn, Dimpy P. Shah, Susanne Schmidt, Yui‐Wing F. Lam, Daniel MacCarthy, Laura Tenner, Amelie G. Ramirez

**Affiliations:** ^1^ Department of Epidemiology and Biostatistics University of Texas Health Science Center at San Antonio (UTHSCSA) San Antonio Texas; ^2^ Department of Pharmacology UTHSCSA San Antonio Texas; ^3^ Institute for Health Promotion Research, UTHSCS San Antonio Texas; ^4^ Mays Cancer Center UTHSCSA San Antonio Texas

**Keywords:** disparity, hepatocellular carcinoma, metformin, type 2 diabetes

## Abstract

**Background:**

We assessed racial/ethnic disparity in hepatocellular carcinoma (HCC) incidence among men with type 2 diabetes (T2D) but without chronic liver diseases (CLD), and whether metformin use modified the disparity.

**Methods:**

Study cohort: the nationwide Veterans Administration Health Care System electronic medical records among 40‐89 years old men with T2D; without CLD, cancer, cardiovascular or renal diseases previously; insulin and thiazolidinedione naive. Logistic regression analyses compared HCC incidence between race/ethnicity groups under no metformin use adjusted for covariates and inverse propensity score weights (IPSW) for race/ethnicity. The generalizability technique integrated with IPSW was incorporated to compare covariates adjusted odds ratios (aOR) of HCC associated with metformin use among race/ethnicity groups.

**Results:**

Study cohort: N = 84 433; 79.47% non‐Hispanic white (NHW), 15.5% non‐Hispanic African American (NHAA), 5.03% Hispanics; 36.76% metformin users; follow‐up 6.10 ± 2.87 years; age 67.8 ± 9.8 years, HbA1c 6.57 ± 0.98%; 0.14% HCC cases. Under no metformin use, HCC incidence was lower for NHAA vs NHW (aOR = 0.60 [0.40‐0.92]), similar between NHW and Hispanics. Metformin was associated with reduced HCC risk: aOR = 0.57 (0.40‐0.81) for NHW; aOR = 0.35 (0.25‐0.47) for NHAA; aOR = 0.31 (0.22‐0.43) for Hispanics. Metformin dose >1000 mg/d was neutral for NHW; less effective for NHAA (*P* = 0.02); more effective for Hispanics (*P* = 0.002).

**Conclusions:**

In men with T2D but without CLD nor metformin use, HCC incidence was lower for NHAA compared to NHW or Hispanics; similar between NHW and Hispanics. Metformin use reduced HCC risk and modified the race/ethnicity disparity.

**Impact:**

Metformin's heterogeneous HCC prevention effect elucidates potential interventions to modify HCC disparity in patients with T2D.

## 1 INTRODUCTION

Among significant contributors to hepatocellular carcinoma (HCC), type 2 diabetes (T2D) and chronic liver diseases (CLD) are subject to remarkable racial/ethnic variations.^1^, ^2^, ^3^, ^4^, ^5^, ^6^, ^7^, ^8^, ^9^, ^10^, ^11^, ^12^ Notably, some study design strategy has been employed to investigate the racial/ethnic disparity in HCC attributed to or independent of T2D by isolating the disparity associated with CLD.^10^, ^11^, ^12^, ^13^ For example, two studies among individuals with hepatitis C virus (HCV) showed that compared to non‐Hispanic white (NHW), Hispanics had a significantly higher risk for HCC that was independent of T2D, whereas African American (AA) had a substantially lower risk of developing cirrhosis and HCC.^10^, ^11^ A similar design strategy was also used to verify T2D as an independent risk for HCC among individuals not infected with hepatitis B virus or HCV.^12^, ^13^ The HCC disparity inferred from these study populations with CLD however may not be generalizable to individuals without CLD. In particular, the increased HCC risk associated with T2D was found to be more pronounced for individuals without CLD compared to those with CLD.^13^

Metformin remains the mainstay first‐line medication for treating T2D, and it has demonstrated HCC prevention effect^14^ via regulating hepatic steatosis,^15^ and inhibiting the growth of HCC tumors.^16^ In addition, the heterogeneous effects of metformin by race‐ethnicity as evident in the literature 17, 18, 19, 20, 21, 22 could play a role on modifying the racial/ethnic disparity in HCC. In particular, two lines of research pointed out that: (a) Hispanics are more likely to carry the allele of the ethnic‐specific transporter that is associated with retention of metformin in the renal.^17^, ^18^, ^19^; (b) better metformin response is often observed in race/ethnic populations who are more prone to metabolic syndrome or chronic inflammation.^20^, ^21^, ^22^

This study assessed the racial/ethnic disparity in HCC incidence among men with T2D but without CLD nor use of metformin as well as whether this disparity in HCC could be altered by metformin use. We address these research questions using a historical longitudinal cohort among the nationwide male veterans with T2D but without CLD. To quantify HCC disparity in concordance with the Institute of Medicine (IOM; currently called the National Academy of Medicine) definition as well as to enhance the causal inference of the metformin effect, we integrated the propensity score weighting technique^23^ and the generalizability method^24^ in our statistical analyses to calibrate between race/ethnic groups and minimize confounding.

## 2 MATERIALS AND METHODS

### 2.1 Study cohort

The historical longitudinal cohort for this study was derived from the electronic medical records (EMR) in the nationwide Veterans Administration Health Care System (VAHCS) databases during fiscal year (FY) 2001 and FY2012. Inclusion criteria were men of 40‐89 years old in FY2003; with any diagnosis of T2D during FY2001‐FY2002 as well as in FY2003; without prescriptions for T2D medications nor any diagnosis for cancer, renal, chronic liver, or cardiovascular diseases (CVD) during FY2001‐FY2002. For the purpose of this study, patients with prescriptions for insulin or thiazolidinedione during FY2003‐FY2012 were excluded to eliminate these drugs’ effects on cancer incidence or progression as reported previously.^25^ We further limited the study cohort to 84 433 patients without any missing covariates. This study was approved by the Institutional Review Board of the University of Texas Health Science Center at San Antonio.

### 2.2 Data sources

Patient level variables were derived from linked VAHCS datasets between October 1 2001 (the beginning of FY2001) and September 30, 2012: Inpatient and Outpatient Medical SAS Datasets, the VA Decision Support System, and the Corporate Data Warehouse.^26^ Patients’ neighborhood social economic status (SES) variables were obtained from the American Community Survey (ACS, ref. 27) by linking patient's zip codes to the 5‐years estimate of educational attainment from ACS 2007‐2011, and the 5‐years estimate of poverty and health insurance from ACS 2008‐2012. The ACS datasets were chosen to strike a balance between completeness, currency, and precision of the SES data.

### 2.3 Outcomes of interest

The outcome of interest in this study was the incidence of any HCC diagnosis (ICD‐9 diagnosis of 155.XX) during the study period. The study starting date was the first date of FY2003 for patients without any glucose‐lowering medication prescription, the initiation date of non‐metformin glucose‐lowering medication for patients with non‐metformin glucose‐lowering medication prescriptions, and the initiation date of metformin for metformin users. The study termination date of a patient was the earliest between the date of first HCC diagnosis, the date of death, and September 30, 2012.

### 2.4 Metformin exposure

In the primary analysis, metformin use was defined as ≥180 days of prescription at any dose, an exposure cut‐point commonly used in clinical research.^28^, ^29^ Non metformin users were those without any prescription for metformin during the study period. We also conducted secondary analyses to assess the dose‐response effect of metformin use, where HCC incidence associated with an average dose of ≥1000 mg/d was compared to <1000 mg/d. This dose‐response analysis was conducted under ≥90, ≥120, or ≥180 days of prescription for metformin to examine the trend in exposure intensity/length.

### 2.5 Race/ethnicity

Three race/ethnic groups were examined: Hispanics, non‐Hispanic AA (NHAA), and NHW.

### 2.6 Covariates

Covariates adjusted for in the analyses included age; age‐adjusted Charlson co‐morbidity score^30^; statin use^31^, ^32^, ^33^; beta‐blocker use^34^; baseline and temporal change of body mass index (BMI), low‐density lipoprotein (LDL) and hemoglobin A1c (HbA1c); having had diagnosed for alcohol related mental health disorders (V40.2 V40.9 294.8 294.9); nonspecific abnormal liver functions (794.8) during the study period; and patient's residential neighborhood SES (including the proportion of households in poverty, the proportion of individuals with health insurance, the proportion of adults of ≥25 years old with a completed high school education at the zip code level).

### 2.7 Statistical analyses

#### 2.7.1 Racial/ethnic disparity in HCC incidence under no use of metformin

Due to the low incidence of HCC observed in this study, logistic regression model was used for analyses of HCC incidence in association with race/ethnicity among patients without metformin use. Predictors in the analysis of HCC incidence included indicators of NHAA and Hispanics (NHW being the referent), study duration, age, comorbidity, indicators of statin use^31^, ^32^, ^33^ and beta‐blocker use,^34^ change in LDL, HbA1c, and BMI, alcohol‐related mental health disorders, abnormal liver functions, and neighborhood SES. To infer the racial/ethnic disparity in HCC incidence that is in concordance with the NAM's definition, the inverse propensity scores^23^, ^24^, ^29^ of race/ethnicity were incorporated as the weights in the logistic regression model to calibrate between race/ethnic groups. The predictors for propensity scores of race/ethnicity group membership included baseline age, neighborhood SES, HbA1c, BMI, LDL, statin use, beta‐blocker use, and Charlson comorbidity score. The adjusted odds ratio (aOR) associated with NHAA or Hispanic relative to NHW derived from the inverse propensity score weights (IPSW) adjusted analysis projected the racial/ethnic difference in HCC incidence should the baseline covariates be balanced between racial/ethnic groups. All model parameters were assessed by the Wald test with *P* < 0.05 being significant.

#### 2.7.2 Differential metformin effect on HCC incidence by race/ethnicity

Logistic regression analyses of both metformin users and non users were conducted for each race/ethnic group to estimate the race/ethnic specific aOR associated with metformin use, where predictors included metformin use, statin use, and beta‐blocker use, interactions between metformin use with statin/beta‐blocker use, age, study duration, change of LDL, HbA1c, and BMI during the study period, and other covariates. To enhance the causal inference about the association between metformin use with HCC incidence, the IPSW of metformin use was incorporated in the analysis to achieve balance in baseline covariates between metformin users and non‐users^29^: each propensity score was the likelihood of metformin use for each patient conditioned on baseline age, HbA1c, BMI, and Charlson comorbidity score calculated from the logistic regression model. To test the discrepancy of metformin's effect between race/ethnicity groups, we employed the generalizability analysis method 24 to project whether the effect of metformin differed between race/ethnic groups should the post‐baseline clinical characteristics (HbA1c, BMI, LDL, and Charlson comorbidity) be equalized between groups. That is, the generalizability analysis calculated the effect of metformin on HCC incidence for NHAA and Hispanics based on their respective weighted likelihood functions such that the post‐baseline clinical characteristics for NHAA/Hispanics were calibrated as those for NHW, where the weights were the ratios of the proportion of the NHAA/Hispanic group to the propensity scores of being in the NHAA or Hispanic group conditioned on their clinical characteristics.^24^ The effect associated with metformin use derived from the generalizability analysis projected the effect of metformin on HCC incidence for NHAA or Hispanics should the clinical characteristics during the study period be calibrated between race/ethnic groups and the baseline covariates be balanced between metformin users and non‐users. The adjusted ORs of HCC associated with metformin use were derived separately by race/ethnic group based on each group's corresponding weighted likelihood function with the post‐baseline clinical characteristics being calibrated towards NHW and baseline clinical characteristics being balanced between metformin users and nonusers. Subsequently, the difference of aORs between any pair of race/ethnicity groups was verified by two‐sample *t*‐test. A similar approach was used to test the differential aORs associated with covariates between race/ethnic groups. Therefore, the differential aORs between race/ethnicity groups should be interpreted as the projected difference should the post‐baseline clinical characteristics be calibrated towards NHW and baseline clinical characteristics be balanced between metformin users and nonusers. To complement the stratified analysis for assessing the heterogeneous effects between race/ethnic groups as described above, we also conducted analyses of all study subjects that allowed interactions involving race/ethnicity.

To examine the dose‐response effect of metformin, the methods as described above were modified to compare the effect of average metformin daily dose (≥1000 mg vs <1000 mg) among metformin users stratified by the length of metformin exposure: ≥90, ≥120, or ≥180 days of prescription. In these analyses, the IPSWs of higher average metformin daily dose (≥1000 mg/d) were incorporated as weights in the logistic regression analyses of HCC to hypothetically equalize the baseline characteristics between metformin users of high and low doses.

The potential estimation bias associated with low HCC incidence was assessed by sensitivity analysis.^35^ All statistical analyses were conducted using SAS 9.3.

## 3 RESULTS

This study cohort consisted of 84 433 men with T2D but without prior CLD with mean follow‐up 6.10 ± 2.87 years, 67 065 (79.47%) NHW, 13 125 (15.5%) NHAA, 4243 (5.03%) Hispanics, mean age 67.8 ± 9.8 years, mean HbA1c 6.57 ± 0.98%, 121 (0.14%) diagnosed for HCC, and 31 036 (36.76%) metformin users. Table 1 showed significant differences in subjects’ characteristics at baseline between metformin users and non‐users as well as between race/ethnic groups, which included predictors for HCC, such as age, BMI, and medication use. Thus, we estimated the effects associated with race/ethnicity and metformin use by incorporating the IPSW for race/ethnicity membership or metformin use to eliminate potential confounding.

**Table 1:** Study cohort (n = 84 433) characteristics by race/ethnicity and by metformin use

	NHW (n = 67 065)		NHAA (n = 13 125)			Hispanic (n = 4243)			Metformin users (n = 28 716)		Non metformin users (n = 53 397)		
	Mean (%)	SD	Mean (%)	SD		Mean (%)	SD		Mean (%)	SD	Mean (%)	SD	
HCC (%)	0.13		0.14			0.15			0.07		0.17		b
Statin use (%)	72.01		68.86		a	75.92		a	84.46		64.44		b
Beta‐blocker use (%)	54.56		53.19		a	48.03		a	52.54		55.10		b
Sulfonylureas use (%)	59.05		58.78		a	62.21		a	75.71		49.45		b
Age (y)	69.83	9.55	64.74	10.83	a	67.26	9.83	a	65.38	9.59	71.17	10.24	b
Charlson comorbidity	3.63	2.6	3.83	2.85	a	3.61	2.65	a	3.36	2.33	3.83	2.91	b
ΔA1C (%)	0.05	0.83	‐0.1	1.02	a	0.06	0.89		0.07	0.94	0.02	0.87	b
ΔBMI (kg/m^2^)	‐1.08	3.28	‐1.08	2.12	a	‐1.02	1.88		‐1.16	1.77	‐1.05	2.00	b
ΔLDL (mg/L)	‐16.81	24.62	‐17.94	28.02	a	‐18.69	23.34	a	‐18.94	22.69	‐15.97	24.81	b
Baseline A1C (%)	6.58	0.95	6.79	1.21	a	6.62	0.99	a	6.92	1.00	6.42	1.03	b
Baseline BMI (kg/m^2^)	30.94	7.01	31.11	5.87	a	29.22	4.87	a	31.37	4.93	30.60	5.07	b
Baseline LDL (mg/L)	104.39	26.69	111.32	29.82	a	107.14	25.92	a	104.08	24.69	106.17	27.64	b
Neighborhood poverty (%)	10.74		10.69			10.83			10.74		10.74		
Neighborhood High School completion (%)	84.53		84.58			84.38			84.52		84.54		
Neighborhood Health insurance coverage (%)	64.40		64.51			64.45			64.39		64.43		
Alcohol related mental health disorder (%)	4.82		4.99			4.76			4.81		4.87		
Nonspecific abnormal liver functions (%)	0.13		0.14			0.15			0.07		0.17		

BMI, body mass index; HCC, hepatocellular carcinoma; LDL, low‐density lipoprotein; NHAA, non‐Hispanic African American; NHW, non‐Hispanic white.

a Significant difference between NHAA/Hispanics and NHW.

b Significant difference between metformin users and nonusers.

### 3.1 Race/ethnic difference under no use of metformin

Among patients with T2D but without CLD nor metformin use, the NAM concordant disparity measures showed that the HCC incidence was 40% lower for NHAA compared to NHW (aOR = 0.60, 95% CI = [0.40, 0.96], *P* < 0.001), but similar between NHW and Hispanic (aOR = 0.95, 95% CI = [0.40, 2.24], *P* = 0.55).

### 3.2 Effects of metformin

In the analysis of the entire cohort adjusted for covariates and propensity of metformin use, it showed that metformin use was associated with an overall 51% decreased HCC risk across all race/ethnic groups (aOR = 0.49, 95% CI = [0.36, 0.66]). When further applying the generalizability method to calibrate covariates between race/ethnic groups, we found that metformin's HCC prevention effect would have been superior for Hispanics and NHAA compared to NHW: aOR associated with metformin use was 0.57 (95% CI = [0.40, 0.81], *P* = 0.04) for NHW, 0.35 (95% CI = [0.25, 0.47], *P* < 0.001) for NHAA, and 0.31 (95% CI = [0.22, 0.43], *P* < 0.001) for Hispanics; the estimated metformin effect for Hispanics and NHAA differed significantly from that for NHW (*P*‐values are 0.007 and 0.02, respectively). As shown in Table 2, the dose‐response effect of metformin also differed by race/ethnicity. Among NHW, the HCC prevention effect associated with metformin use did not differ by the average daily dose (≥1000 mg/d vs <1000 mg/d) regardless of days of prescription. In contrast, the HCC prevention effect among NHAA was attenuated under a higher average daily dose of metformin use, while higher average daily dose of metformin among Hispanics was associated with a greater HCC prevention effect. The aOR's associated with an average of ≥1000 mg/d (vs <1000 mg/d) for NHAA were 3.48 (*P* = 0.001), 2.68 (*P* = 0.02), and 2.76 (*P* = 0.02) under ≥90, ≥120, and ≥180 days of prescription of metformin; the corresponding aOR's among Hispanics were 0.11 (*P* = 0.004), 0.07 (*P* = 0.002), 0.07 (*P* = 0.002).

**Table 2:** AOR^a^ of HCC diagnosis associated with metformin daily dose ≥1000 mg (vs daily dose <1000 mg) stratified by race/ethnicity among metformin users with ≥90, ≥120, or ≥180 d of prescription

	NHW		NHAA		Hispanic	
	aOR (95% CI)	*P*‐value	aOR (95% CI)	*P*‐value	aOR (95% CI)	*P*‐value
≥90 d	0.91 (0.71‐1.16)	0.44	1.99 (0.88‐4.51)	0.77	0.04 (0.01‐0.17)	<0.01
≥120 or ≥180 d	0.97 (0.69‐1.36)	0.84	2.43 (1.04‐5.71)	0.04	0.02 (0.002‐0.13)	0.01

aOR, adjusted odds ratios; HCC, hepatocellular carcinoma; NHAA, non‐Hispanic African American; NHW, non‐Hispanic white.

a Adjusted for covariates, and propensity scores of metformin daily dose ≥ 1000 mg.

The potential estimation bias associated with small HCC incidence assessed by sensitivity analysis^35^ showed that the potential bias in aOR associated with metformin and race/ethnicity difference were 0.005 and 0.01, respectively. In summary, our data suggested that the race/ethnic disparity in HCC incidence could be modified by metformin use, and that the dose‐response effect of metformin varied by race/ethnicity.

### 3.3 Covariate effects by race/ethnicity

**Figure 1:**
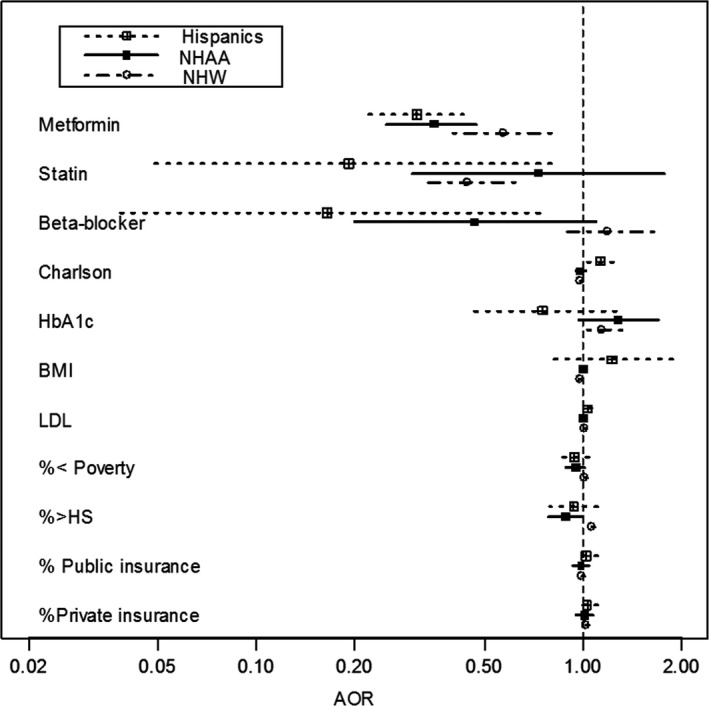
AORs associated with HCC by race/ethnicity (aOR associated with metformin use was adjusted for covariates, inverse propensity scores weighting of metformin use for each race/ethnic group, and inverse propensity scores weighting of race/ethnicity group membership). aORs, adjusted odds ratios; HCC, hepatocellular carcinoma

Significant covariates for HCC incidence included statin use (aOR = 0.44 [*P* < 0.0001] for NHW; aOR = 0.19 [*P* = 0.02] for Hispanics), beta‐blocker use (aOR = 0.16 [*P* = 0.02] for Hispanics), older age (aOR = 1.02 [*P* = 0.02] for NHAA; aOR = 1.13 [*P* = 0.01] for Hispanics), comorbidity (aOR = 1.35 [*P* < 0.0001] for NHW; aOR = 1.56 [*P* < 0.0001] for NHAA; aOR = 1.60 [*P* < 0.0001] for Hispanics), BMI (aOR = 0.98 [*P* = 0.002] for NHW), LDL (aOR = 1.013 [*P* < 0.0001] for NHAA), nonspecific abnormal liver functions (aOR = 5.22 [*P* = 0.002] for NHAA), alcohol related disorder (aOR = 7.01 [*P* < 0.0001] for Hispanics, and neighborhood poverty (aOR = 1.06, [*P* < 0.0001] for NHW) (see Figure 1). The aOR associated with the interaction of metformin with statins use was not significant (*P* = 0.763), indicating no synergistic effect associated with HCC prevention.

The majority of the results regarding differential effects between race/ethnic groups were consistent between the stratified analyses and the model with interactions involving race/ethnicity. Discrepancies between the two approaches were seen in the associations between HCC with Charlson comorbidity score, BMI, LDL, and neighborhood poverty (see the footnotes in Table 3). Compared to the model with interactions, the stratified analysis was more efficient in detecting the differential effect of LDL between NHAA and Hispanics. This discrepancy, according to the simulations by Behrens and colleagues,^36^ could be explained by the effects of opposite directions between race/ethnic groups and a nearly null or weak effect in NHAA. The stratified analyses were also more efficient for detecting differential effects of beta‐blocker use, Charlson comorbidity score, and BMI between race/ethnicity groups. This could be due to the stronger effect in Hispanics or NHAA (the minority) vs a nearly null or weak effect in NHW (the majority).^36^ However, stratified analyses were less efficient for detecting differential effect of neighborhood poverty between Hispanics and NHW. We reported the differential effects between race/ethnic groups primarily based on stratified analyses that permitted more efficient estimates while avoiding potentially obscured results associated with using model with interactions.

**Table 3:** AORs associated with HCC by race/ethnicity (aOR associated with metformin use was adjusted for covariates, inverse propensity scores weighting of metformin use for each race/ethnic group, and inverse propensity scores weighting of race/ethnicity group membership)

	NHW	NHAA	Hispanics	*P*‐values for difference between race/ethnicity groups		
	aOR (95% CI)	aOR (95% CI)	aOR (95% CI)	NHAA/NHW	Hispanics/NHW	Hispanics/NHAA
Metformin	0.570 (0.400, 0.810)	0.350 (0.250, 0.470)	0.310 (0.220, 0.430)	0.025	0.008	0.31
Statin use	0.442 (0.317, 0.616)	0.727 (0.298, 1.770)	0.192 (0.046, 0.794)	0.152	0.131	0.06
Beta blocker use	1.185 (0.857, 1.637)	0.466 (0.199, 1.089)	0.164 (0.037, 0.734)	0.022^a^	0.006^a^	0.118
Age (y)	1.019 (1.003, 1.034)	1.019 (0.984, 1.055)	0.884 (0.807, 0.969)	0.50	0.001	0.002
Charlson comorbidity	0.981 (0.967, 0.997)	0.981 (0.948, 1.016)	1.131 (1.032, 1.239)	0.50	0.001^a^	0.002
ΔA1C (%)	1.140 (0.989, 1.316)	1.277 (0.964, 1.692)	0.750 (0.446, 1.261)	0.24	0.064	0.039
ΔBMI (kg/m^2^)	0.977 (0.962, 0.991)	0.996 (0.984, 1.008)	1.220 (0.796, 1.869)	0.024^a^	0.154	0.176
ΔLDL (mg/L)	1.013 (1.007, 1.019)	0.997 (0.987, 1.007)	1.026 (0.995, 1.058)	0.003	0.219	0.041^a^
Neighborhood poverty (%)	1.005 (0.980, 1.031)	0.946 (0.886, 1.010)	0.938 (0.848, 1.037)	0.046	0.096^b^	0.445
Neighborhood High School completion (%)	1.062 (1.036, 1.088)	0.883 (0.780, 1.000)	0.933 (0.789, 1.103)	0.002	0.067	0.304
Neighborhood public Health insurance coverage (%)	0.992 (0.971, 1.013)	0.982 (0.933, 1.035)	1.016 (0.935, 1.105)	0.365	0.293	0.249
Neighborhood private Health insurance coverage (%)	1.016 (0.997, 1.036)	1.008 (0.951, 1.070)	1.025 (0.952, 1.101)	0.399	0.416	0.367

aOR, adjusted odds ratios; BMI, body mass index; HCC, hepatocellular carcinoma; LDL, low‐density lipoprotein; NHAA, non‐Hispanic African American; NHW, non‐Hispanic white.

a Model with interactions involving race/ethnicity suggested nonsignificant interaction effect.

b Model with interactions involving race/ethnicity suggested significant interaction effect.

## 4 DISCUSSIONS

In this cohort of 84 433 nationwide male veterans with T2D but without CLD who were insulin and thiazolidinedione naïve, we found that under no use of metformin, NHAA was associated with a 40% reduction in HCC incidence compared to NHW or Hispanic men should baseline clinical characteristics be calibrated between race/ethnic groups. A similar HCC incidence was found between NHW and Hispanics. The lower HCC incidence in NHAA found in this study was consistent with the literature. However, unlike the ethnic disparity in HCC reported in prior studies with a mixed CLD and T2D statuses, we found a similar HCC incidence between Hispanic and NHW men with T2D but without CLD. The contrast of prior and present findings suggested that in the absence of CLD, T2D may alter the ethnic disparity in HCC. This longitudinal study is also the first to show that the superior HCC prevention effect associated with metformin use for Hispanics and NHAA compared to NHW, and differential dose‐response effect of metformin between race/ethnic groups. Although the effects of statin and beta‐blocker use on HCC risk appeared to differ by race/ethnicity, they did not modify metformin's HCC prevention effect. Together these results suggested that metformin use could modify race/ethnic disparity in HCC incidence independent of statin and beta‐blocker use.

Our finding of a greater HCC prevention effect associated with metformin use in Hispanics or NHAA compared to NHW could reflect the emerging evidence about heritability of metformin response seen in clinical studies,^37^, ^38^ or ethnic difference in allele frequency associated with metformin transporters that affect metformin's bioavailability shown in preclinical studies.^17^ The modulatory effect of metformin as a peroxisome proliferator‐activated receptor gamma agonist on the highly prevalent PNPLA3 I148M variant in Hispanics could also play a role.^39^ Thus pharmacogenomics of metformin response in terms of HCC prevention warrants further investigation as a plausible explanation for the differences observed.

Among predictors for HCC incidence, our data showed that older age, comorbidity, increased LDL, and alcohol related mental health disorder were associated with increased HCC incidence, while use of statins or beta‐blockers was associated with decreased HCC incidence. However, it is not clear about the null association between HbA1c and HCC in this cohort. The variation of covariate effects by race/ethnicity suggested race/ethnic sensitive management of these factors for HCC prevention in men with T2D. Metformin could affect HbA1c, BMI, or LDL, and the better metformin response regarding HCC prevention might be mediated through HbA1c, BMI or LDL. However, the variable availability of longitudinal assessments of these clinical factors at the patient level from the VAHCS EMR may not be ideal to evaluate these mediation effects.

Similar to prior studies,^31^ we also found that use of statins was associated with decreased HCC incidence. However, since no patients in this cohort had HCV, our finding suggested that the effect of statin on HCC could be independent of its inhibition of the replication of HCV,^32^ or the response to antiviral therapy (eg, peginterferon and ribavirin).^33^ In this cohort, use of beta‐blockers was associated with reduced HCC incidence only among Hispanics. However, the beta‐blocker type was not available to examine whether the ethnic difference was due to differential use of non‐selective beta‐blockers.^40^

We note the limitations of this observational study design using EMR and the associated remedies. First, several potential factors associated with the disparity in HCC were not accessible, including T2D duration, the distribution and composition of the adipose tissues,^41^, ^42^ and exposure to non‐pharmacological treatments for T2D. Thus, the similarity of T2D profile at baseline across three race/ethnic groups were limited to being insulin naïve, under reasonable glycemic control, and without a history of cancer, CVD, and CLD. While the relationship between individual level BMI and HCC is well‐established yet complex,^43^ our data from EMR however fell short to reveal a clinically significant association between BMI with HCC nor the variation of BMI between race/ethnic groups. This could have an implication on the extent to which the confounding associated BMI could be addressed. This limitation also applies to other anthropometric measures that could inform adipose distribution yet often available in a more skewed subpopulation.^44^ Nevertheless, we have managed to strengthen the causal inference derived from this non‐randomized study by incorporating the IPSW technique to minimize confounding by calibrating covariates (including on age, HbA1c, BMI, and Charlson comorbidity score) between comparison groups. In addition, to ensure robust inference derived from this study in the presence of small HCC incidence, sensitivity analysis was conducted to assess the potential estimation bias associated with low HCC incidence. Furthermore, although the study cohort only included men, this also bears the strength to curtail the disparity associated with sex hormones, such as estrogen which may exert protective effects against HCC through Interleukin‐6 restrictions.^45^ The findings of heterogeneous metformin response among racial/ethnic groups appeared to be robust even after accounting for the potential estimation bias associated with small HCC incidence.

In conclusion, our study showed that in men with T2D but without CLD, racial/ethnic disparity in HCC incidence was modified by metformin use. These results should be further verified by translational research using cohorts with adequate representation of the minority population that combines both clinical trials to assess race/ethnic specific effects of metformin and mechanistic studies to examine the potential effect of ethnic‐specific genetic variation on drug response. Given the rising T2D epidemics, further investigating HCC prevention effects of otherT2D treatments and their impacts on race/ethnic disparity in HCC risk could help identify tailored clinical management for HCC prevention in men with T2D.

## CONFLICT OF INTEREST

None declared.
